# Allopurinol and global left myocardial function in heart failure patients

**DOI:** 10.4103/0975-3583.74262

**Published:** 2010

**Authors:** Gamela Nasr, Cherine Maurice

**Affiliations:** *Department of Cardiology, Suez Canal University, Ismailia, Egypt*; 1 *Department of Pharmacology, Suez Canal University, Ismailia, Egypt*

**Keywords:** Allopurinol, congestive heart failure, Tei index, uric acid

## Abstract

**Background and Aim:**

Increased xanthine oxidase (XO) activity may contribute to heart failure pathophysiology. This study evaluated whether a XO inhibitor, allopurinol produces clinical and functional benefits in patients with New York Heart Association functional class III to IV heart failure due to systolic dysfunction receiving optimal medical therapy as estimated by global left myocardial function.

**Patients and Methods:**

Fifty-nine patients with a diagnosis of chronic heart failure due to coronary heart disease or idiopathic dilated cardiomyopathy and 20 healthy controls who attended the outpatient clinic of cardiology were subjected to full echocardiographic study including left ventricular diastolic and systolic function, and the combined index of myocardial performance [Tei index: isovolumetric relaxation time (IRT) + isovolumetric contraction time (ICT)/ejection time (ET)]. Patients were randomized to allopurinol (300 mg/day) or placebo. Improvement at 36 weeks was assessed using a composite end point comprising global left cardiac function as well as heart failure morbidity and mortality.

**Results:**

The percentage of patients characterized as improved, unchanged, or worsened did not differ between those receiving allopurinol or placebo. Allopurinol reduced serum uric acid (SUA) by 1.5 mg/dL (*P* = 0.001). In a subgroup analysis, patients with elevated SUA (more than 7mg/ dL) responded favorably to allopurinol whereas those with SUA less than 7mg/dL exhibited a trend toward no change. In addition, SUA reduction to allopurinol correlated with favorable clinical and functional response. Within the entire allopurinol patient cohort, those characterized as either improved or unchanged had significantly greater reductions in SUA compared with patients who did not change (*P* = 0.0007). In placebo patients, lower baseline SUA, but not change in SUA, correlated with improved clinical outcome.

**Conclusions:**

Allopurinol did not produce significant clinical and functional improvement in unselected patients with moderate-to-severe heart failure. However, it is suggested that it is useful in patients with elevated SUA in a manner according to degree of SUA reduction. SUA may serve as a valuable biomarker to target heart failure therapy.

## INTRODUCTION

Most epidemiological studies associate increasing serum uric acid (SUA) with increased cardiovascular event rate and mortality in those with known or elevated risk of vascular disease and among healthy volunteers. These data have been thoroughly reviewed in many studies.[1] There is a strong evidence supporting a pathophysiological role for the xanthine oxidase (XO) pathway in heart failure.[2] From a functional standpoint, increased XO activity causes cardiac mechanoenergetic uncoupling and vascular dysfunction in the failing circulation. Therefore, there is a place to improve cardiac function by its inhibition. The prototypical (XO) inhibitor, allopurinol, has been the cornerstone of the clinical management of gout and conditions associated with hyperuricemia for several decades. More recent data indicate that XO also plays an important role in various forms of ischemic and other types of tissue and vascular injuries, inflammatory diseases, and chronic heart failure (CHF).[3]

Mellin*et al*.[3] report that short- and long-term (XO) inhibition with allopurinol, initiated in rats with CHF due to myocardial infarction, improves cardiac hemodynamics and function and reverses left ventricular (LV) remodeling.

Echocardiography has introduced the possibility to evaluate not only systolic, but also diastolic function. LV systolic dysfunction reduces ejection fraction and ejection time, and prolongs isovolumetric contraction time. On the other hand, LV diastolic dysfunction increases isovolumetric relaxation time (IRT) and modifies the timing of diastolic filling. A relatively new Doppler index of combined systolic and diastolic myocardial performance of the left ventricle, defined as the sum of the isovolumetric contraction time and IRT divided by ejection time, has been reported.[4,5]

This index is easily obtained, reproducible, has a narrow range in normal subjects, does not depend on LV geometry, and correlates with invasive obtained measures of systolic and diastolic cardiac function.[6] This index has been reported to be simple and independent of heart rate and blood pressure.

This study not only raises an interesting suggestion regarding the role of oxidative stress in heart failure, but also offers a new pharmacological tool for a novel class of heart failure therapeutics: the XO inhibitors. This is by the use of a relatively new sensitive Doppler index of combined systolic and diastolic myocardial performance of the left ventricle.

## PATIENTS AND METHODS

Fifty-nine patients with a diagnosis of CHF due to coronary heart disease or idiopathic dilated cardiomyopathy and 20 healthy controls who attended the outpatient clinic of cardiology were subjected to full echocardiographic study including LV diastolic and systolic function, and the combined index of myocardial performance (Tei index = IRT + ICT/ET).[7] Patients were randomized to allopurinol (300 mg/day) or placebo. Improvement at 36 weeks was assessed using a composite end point comprising global left cardiac function as well as heart failure morbidity and mortality.

### Doppler echocardiographic parameters

Patients were subjected to M-mode and two-dimensional (2D) echocardiography with spectral and color flow Doppler analysis using a Hewlett-Packard (model Sonos 1800; Hewlett Packard, Model DR 53 15 Andover, MA) ultrasound machine with a 2.5/3.5-MHz transducer. Patients were examined in left lateral decubitus position. In this study, the following parameters were considered: left ventricular end-diastolic (LVDD) and end-systolic (LVSD) left ventricular diameters; end-diastolic (RVDD) and end-systolic (RVSD) right ventricular diameters, interventricular-septal wall (SWT) and posterior left ventricular wall (PWT) thickness (mm). The systolic function was assessed by the ejection fraction of the left ventricle (EF) in percentage calculated using the area–length method. Diastolic function was evaluated by *E/A* ratio (*E* velocity = early maximal ventricular filling velocity, *A* velocity = late diastolic or atrial velocity), by analysis of trans-mitral (*L*) flow for the assessment of diastolic function.

Left ventricular mass (LVM) was calculated with the application of Devereux’s formula:[8] $\mathrm{LVM}\;=\;1.04\;\times \;\left[\left(\mathrm{LVEDD}\;+\;\mathrm{LVEDSEP}\;+\;\mathrm{LVEDPW}\right)-{\mathrm{LVEDD}}^{3}\right]-13.6.$

### Left ventricular total isovolumic ejection index (Tei index)

The IRT was measured with the pulsed wave sample volume placed between the mitral inflow and the LV outflow tract. The total isovolumic ejection index (Tei index)[9] was obtained as the sum of both contraction and relaxation isovolumetric periods, divided by the ejection time. These Doppler time intervals were measured from mitral inflow and LV outflow velocity spectral signals. Several Doppler measurements were made by an independent observer blinded to patient diagnosis. The cut-point value for the index was considered 0.43 for the left ventricle.[9]

## CONTROLS

Forty control subjects were studied. They were healthy volunteers. The same procedures for the echocardiographic examinations were applied.

### Statistical analysis

Data are expressed as mean ± SE. The statistical significance of the differences between the groups was calculated using Student’s *t*-test for independent means. Two-tailed values of *P* < 0.05 were considered significant.

### Ethical consideration

Informed consent was obtained from all the adults. The aim and the value of the work were explained in a simplified manner for them. There was no harm inflicted on them. On the contrary, all had benefits of the follow-up and the final results of the study.

## RESULTS

The percentage of patients characterized as improved, unchanged, or worsened did not differ between those receiving allopurinol or placebo. Allopurinol reduced SUA by 1.5 mg/dL (*P* = 0.001). In a subgroup analysis, patients with elevated SUA (more than 7mg/dL) responded favorably to allopurinol whereas those with SUA less than 7 mg/dL exhibited a trend toward no change. Within the entire allopurinol patient cohort, those characterized as either improved or unchanged had significantly greater reductions in SUA compared with patients who did not change. In placebo patients, lower baseline SUA, but not change in SUA, correlated with improved clinical outcome.

Regarding clinical improvement, there existed non-significant changes regarding symptoms of heart failure with no significant change in the grade of dyspnea.

Functional echocardiography findings in controls and heart failure patients due to coronary heart disease before and after 36 weeks after receiving allopurinol (300 mg/day) are shown in Table 1. On the other hand, Table 2 shows functional echocardiography findings in controls and heart failure patients due to idiopathic dilated cardiomyopathy before and after 36 weeks of receiving allopurinol (300 mg/day).

**Table 1:** Functional echocardiography findings in controls and heart failure patients due to coronary heart disease before and after 36 weeks after receiving allopurinol (300 mg/day)

Parameters	Groups							
	Control (n = 20)				Heart failure due to coronary heart disease (n = 34)			
	Before		After		Before		After	
	Mean	±SD	Mean	±SD	Mean	±SD	Mean	±SD
EF %	62.7	5.5	62.9	5.3	46.5	3.9	47.5	4.3
Tei index	0.41	3.8	0.41	3.9	0.49	5.4	0.47	3.8
E/A	1.3	0.3	1.2	0.4	0.9	0.3	0.85	0.7

*Significance at <0.05.

**Table 2:** Functional echocardiography findings in controls and heart failure patients due to idiopathic dilated cardiomyopathy before and after 36 weeks of receiving allopurinol (300 mg/day)

Parameters	Groups							
	Control (n = 20)				Heart failure due to idiopathic dilated cardiomyopathy (n = 25)			
	Before		After		Before		After	
	Mean	±SD	Mean	±SD	Mean	±SD	Mean	±SD
EF %	62.7	5.5	62.9	5.3	36.5	3.9	39.5	4.1
Tei index	0.41	3.8	0.41	3.9	0.61	4.8	0.60	3.3
E/A	1.3	0.3	1.2	0.4	0.7	0.55	0.8	0.6

*Significance at <0.05.

## DISCUSSION

Chronic heart failure is characterized by a progressive LV remodeling[10,11] and despite the introduction of newer drugs, it has a poor prognosis.[12] Although the involvement of systemic and cardiac hemodynamics and neurohumoral factors in the progression of CHF are extensively studied and are recognized as therapeutic targets.[13–15]

Oxygen free radical generation has been proposed to be an important mechanism of cellular injury in ischemic and reperfused tissues. Studies in a variety of tissues, including heart, lung, kidney, and brain, have demonstrated that intravascular administration of antioxidant enzymes or free radical-scavenging drugs can prevent reperfusion damage and improve postischemic function.[16]

Although several mechanisms have been demonstrated to be involved in the generation of oxygen free radicals, XO has been proposed to be a central mechanism in a variety of postischemic cells and tissues.[17] Although there has been controversy regarding whether this mechanism occurs in human cells and tissues, recent studies have shown that the enzyme is present in human endothelial cells and is responsible for free radical generation in reoxygenated human endothelial cells.[17]

In patients with idiopathic dilated cardiomyopathy, intracoronary administration of allopurinol resulted in an acute, significant improvement in myocardial efficiency by diminishing oxygen consumption in the presence of standard supportive therapy.[18] CHF also results in an impairment of peripheral vascular reactivity. Acute intravenous infusion of allopurinol or chronic treatment with the drug for 1 month improved endothelial function in patients, as evaluated by the measurement of acetylcholine-induced flow responses.[19]

Some studies have shown that short-term administration of allopurinol improves myocardial efficiency in patients with idiopathic dilated cardiomyopathy by decreasing the oxygen cost of LV contraction.[18] However, this was not evident in this study as allopurinol did not produce significant clinical and functional improvement in unselected patients with moderate-to-severe heart failure. However, it is suggested that it is useful in patients with elevated SUA in a manner according to degree of SUA reduction. This focuses upon the importance of SUA as a valuable biomarker to target heart failure therapy.

In agreement with this, a recent study who demonstrated that oxypurinol did not improve a clinical composite score in patients with systolic, symptomatic heart failure, and could potentially cause harm to some patients. Subgroup analysis supports the concept that patients with these clinical characteristics and a high SUA may benefit from XO inhibition. Moreover, the degree of SUA reduction correlated with clinical outcome, such that patients who worsened despite oxypurinol therapy had relatively less reduction in uric acid. Measurements of uric acid may aid in individualizing therapy with XO inhibitors.[20]

There are several reasons why oxypurinol may not have led to clinical benefits in the broad population of symptomatic, treated patients. First, it is possible that sources of oxidative stress are already inhibited by other medications such as inhibitors of the rennin–angiotensin–aldosterone pathway or carvedilol. In this regard, angiotensin II can activate XO directly[21] and indirectly via NADPH oxidase.[22]

However, more larger studies are needed as XO inhibitor therapy in myocardial infarction and CHF appear appealing possibility for various reasons. First, there is evidence that increased levels of uric acid strongly correlate with mortality rates in congestive heart failure,[23] and XO inhibitors exert certain beneficial effects both in animals and humans with heart failure. Second, allopurinol and its active metabolite oxypurinol are well known and relatively safe drugs that have been used for decades to treat gout. Third, the mechanism of action is unique and thus would be expected to potentiate the beneficial effects of conventional therapeutic agents (e.g., β-blockers and angiotensin-converting enzyme inhibitors).[24]
